# Cushing-Syndrom mit konsekutiver tertiärer Nebennierenrindeninsuffizienz nach simultaner multipler intraartikulärer Lokaltherapie mit Glukokortikoiden: Bei Mehrfachgabe von lokalen Steroiden behandlungsbedürftige Hormonmangelzustände im Blick behalten

**DOI:** 10.1007/s00393-021-01000-8

**Published:** 2021-04-22

**Authors:** A. Schramm, D. Windschall, C. Hinze, D. Föll, H. Wittkowski, H. Winowski, S. Oesingmann

**Affiliations:** 1 Klinik für Kinder- und Jugendmedizin, Allgemeine Pädiatrie, Universitätsklinikum Münster, Münster, Deutschland; 2 Klinik für Kinder- und Jugendrheumatologie, St. Josef-Stift Sendenhorst, Sendenhorst, Deutschland; 3 Klinik für Pädiatrische Rheumatologie und Immunologie, Universitätsklinikum Münster, Domagkstr. 3, 48149 Münster, Deutschland

**Keywords:** Juvenile idiopathische Arthritis, Intraartikuläre Steroidtherapie, Glukokortikoidnebenwirkungen, Nebennierenrindeninsuffizienz, Kortisolmangel, Juvenile idiopathic arthritis, Intra-articular glucocorticoid therapy, Glucocorticoid side effects, Adrenal cortex insufficiency, Cortisol deficiency

## Abstract

Eine 4‑jährige Patientin mit neu diagnostizierter polyartikulärer juveniler idiopathischer Arthritis (JIA) entwickelte nach simultaner multipler Lokaltherapie mit Glukokortikoiden an 46 Stellen zunächst ein Cushing-Syndrom, gefolgt von einer schleichenden Zustandsverschlechterung und schließlich einem akuten hochfieberhaften Harnwegsinfekt. Dabei wurde eine iatrogene Nebennierenrindeninsuffizienz nach der multiplen intraartikulären Glukokortikoidgabe diagnostiziert. Die Möglichkeit schwerer systemischer Glukokortikoidnebenwirkungen nach ausgedehnten Lokaltherapien sollte in das reguläre Management der JIA-Patienten einbezogen werden.

## Abstract (en)

After simultaneous multiple local treatment with glucocorticoids at 46 sites a 4‑year-old female patient with newly diagnosed polyarticular juvenile idiopathic arthritis (JIA) initially developed Cushing’s syndrome followed by a gradual worsening of her condition and finally an acute high fever urinary tract infection. Iatrogenic adrenocortical insufficiency after multiple intra-articular glucocorticoid administration was diagnosed. The possibility of severe systemic glucocorticoid side effects after extensive local treatment should be included in the regular management of JIA patients.

## Anamnese

Bei einer 4 Jahre alten Patientin war die Diagnose einer juvenilen idiopathischen Arthritis (JIA), International League of Associations for Rheumatology(ILAR)-Kategorie Polyarthritis Antinukleäre Antikörper(ANA)-negativ, Rheumafaktor(RF)-negativ), gestellt worden. Die Therapie im kinderrheumatologischen Zentrum bestand zu diesem Zeitpunkt aus einer multiplen lokalen Applikation von Glukokortikoidpräparaten an allen betroffenen Stellen in Narkose, was insgesamt 46 Einzelinjektionen mit einer kumulativen Prednisolon-Äquivalenzdosis von 250 mg erforderte (Tab. 1). Eine systemische Glukokortikoidtherapie erfolgte nicht. Zusätzlich wurde eine Behandlung mit Methotrexat, Ibuprofen und Folsäure p.o. eingeleitet und die Patientin mit Hilfsmitteln und Krankengymnastik versorgt. Die Patientin hatte zunächst gut angesprochen, allerdings nahm sie ca. 2 kg an Gewicht zu (10 %), bei sehr großem Appetit.

**Table 1:** 

Links		Rechts	
Gelenk	Präparat, Dosis	Gelenk	Präparat, Dosis
Schultergelenk	THA 10 mg	Schultergelenk	THA 10 mg
Ellenbogengelenk	THA 10 mg	Ellenbogengelenk	THA 10 mg
Handgelenk radiokarpal	THA 5 mg	Handgelenk radiokarpal	THA 5 mg
Handgelenk mediokarpal	THA 0,5 mg	Handgelenk mediokarpal	THA 0,5 mg
IP-Gelenk	THA 0,5 mg	DS-Gelenk	THA 0,5 mg
MCP I	THA 1 mg	MCP I	THA 1 mg
MCP II	THA 1 mg	MCP II	THA 1 mg
MCP III	THA 1 mg	MCP III	THA 1 mg
MCP IV	THA 1 mg	MCP IV	THA 1 mg
MCP V	THA 1 mg	MCP V	THA 1 mg
PIP II	THA 0,5 mg	PIP II	THA 0,5 mg
PIP III	THA 0,5 mg	PIP III	THA 0,5 mg
PIP IV	THA 0,5 mg	PIP IV	THA 0,5 mg
PIP V	THA 0,5 mg	PIP V	THA 0,5 mg
Hüftgelenk	THA 20 mg	Hüftgelenk	THA 20 mg
Kniegelenk	THA 20 mg	Kniegelenk	THA 20 mg
Oberes Sprunggelenk	THA 10 mg	Oberes Sprunggelenk	THA 10 mg
Talonavikulargelenk	THA 5 mg	Talonavikulargelenk	THA 5 mg
ST-Gelenk	THA 5 mg	ST-Gelenk	THA 5 mg
CN-Gelenk	THA 5 mg	CN-Gelenk	THA 5 mg
Handgelenkstrecksehnen	DEX 1,5 mg	Handgelenkstrecksehnen	DEX 1,5 mg
Tibialis-posterior-Sehne	DEX 1,5 mg	Tibialis-posterior-Sehne	DEX 1,5 mg
Peroneus-brevis-Sehne	DEX 1,5 mg	Peroneus-brevis-Sehne	DEX 1,5 mg
*Kumulative Prednisolon-äquivalente Dosis*^*a*^	*125* *mg*	*–*	*125* *mg*

*CN-Gelenk* Cuneonavikulargelenk, *DEX* Dexamethason, *DS-Gelenk* Daumensattelgelenk, *IP-Gelenk* Interphalangealgelenk, *MCP* Metakarpophalangealgelenk, *PIP* proximales Interphalangealgelenk, *PDN* Prednisolon, *ST-Gelenk* Subtalargelenk, *THA* Triamcinolon-Hexacetonid

^a^Gerundete Prednisolon-äquivalente Dosis in mg gemäß Glukokortikoid-Äquipotenz: Triamcinolon 1 mg = Prednisolon 1,25 mg (75 % Triamcinolon-Anteil in Triamcinolon-Hexacetonid); Dexamethason 1 mg = 6,25 mg Prednisolon

Die Patientin kam 2,5 Monate später zu einem vereinbarten stationären Kontrolltermin. Klinisch bestanden bei Aufnahme keine Arthritis und keine Bewegungseinschränkungen, arthrosonographisch wurde im rechten Sprunggelenk und in einigen Fingergelenken noch Restaktivität gesehen. Nach zunächst stabiler Episode hatte die Patientin im Vorfeld ca. 3 Wochen nach der intraartikulären Behandlung einen zunehmend cushingoiden Habitus entwickelt. Intermittierend traten Erbrechen und Abgeschlagenheit auf. Zehn Wochen nach der initialen Gelenkbehandlung und 10 Tage vor der oben genannten Aufnahme war durch die Kinderärztin im Vorfeld bereits eine Labordiagnostik initiiert worden, dabei war Kortisol im Serum (< 0,05 µg/dl; Normbereich 4,8–19,5 µg/dl) und Urin (< 0,2 µg/l; Normbereich 7–60 µg/l) bei morgendlicher Probenentnahme um 9:00 Uhr nicht messbar. Die Patientin stellte sich schließlich mit Fieber vor, und bei Aufnahme zeigte sie einmalig Erbrechen und einen reduzierten Allgemeinzustand. Das Körpergewicht lag bei 20,9 kg (91. Perzentile), die Körperlänge bei 106,5 cm (72. Perzentile) und der Blutdruck bei 96/58 mm Hg (mittlerer arterieller Druck [MAD] 71 mm Hg). Im Labor zeigten sich erhöhte Entzündungswerte sowie eine Leukozyturie und Proteinurie. Neben einem Harnwegsinfekt wurde ein endokrinologisches Problem als Ursache der Beschwerden vermutet und eine stationäre Abklärung in der Universitäts-Kinderklinik eingeleitet.

## Befund

**Figure 1:**
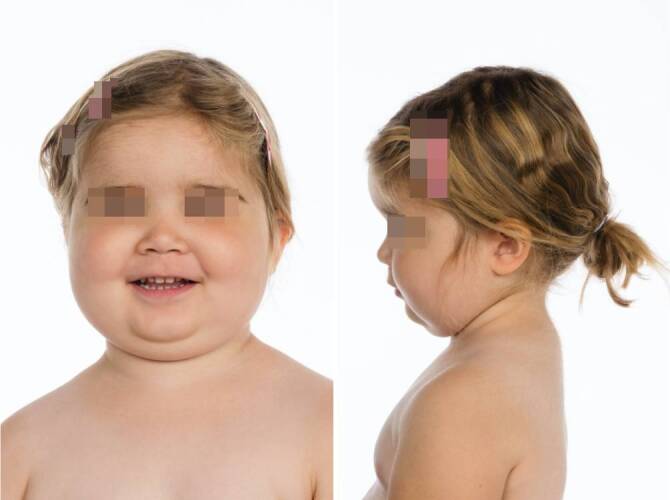

Bei Aufnahme in der Universitätsklinik fielen ein reduzierter Allgemeinzustand mit hohem Fieber und ein adipöser Ernährungszustand bei cushingoidem Habitus auf (Abb. 1). Ein spezifischer Infektfokus war klinisch zunächst nicht eruierbar.

Laborchemisch zeigten sich eine Leukozytose (15.630/µl), Thrombozytose (416.000/µl) sowie eine Erhöhung des C‑reaktiven Proteins (10,2 mg/dl) als Ausdruck einer deutlichen Akute-Phase-Reaktion. Im Urin ließen sich eine Leukozyturie sowie kulturell *E. coli* nachweisen (Keimzahl 10^6^/ml). Die weitere infektiologische und immunologische Diagnostik blieb unauffällig, ebenso blieben Sonographien von Abdomen und Nieren ohne Hinweis auf eine Harntransportstörung.

Die vorstationären Laborbefunde zeigten Konzentrationen von Kortisol sowohl im Serum als auch Urin unterhalb der Nachweisgrenze trotz des bestehenden Cushing-Syndroms. Am Morgen (8:00 Uhr) des zweiten Tages war Kortisol basal mit 141 ng/ml (Norm 11,5–97,5) erhöht und Adrenocorticotropes Hormon (ACTH) lag mit 45,1 pg/ml (Norm 7,3–63,3) im Normbereich. Am Nachmittag (16 Uhr) stieg das ACTH auf 110 pg/ml, um Mitternacht fielen Kortisol (54 ng/ml) und ACTH (2,0 pg/ml) im Rahmen der zirkadianen Rhythmik ab. Die übrige endokrinologische Diagnostik (Schilddrüse, Wachstums- und Sexualhormone) blieb unauffällig, der morgendliche Nüchternblutzucker lag mit 94 mg/dl im Normbereich.

## Diagnose

Es wurde ein hochfieberhafter Harnwegsinfekt mit *E. coli* diagnostiziert. Außerdem bestand ein eindrucksvolles Cushing-Syndrom, vermutlich iatrogen nach intraartikulären Glukokortikoidinjektionen. In Zusammenschau mit den vorstationär nicht nachweisbaren Kortisolspiegeln im Urin und Serum wurde retrospektiv eine konsekutive (tertiäre) Nebennierenrindeninsuffizienz (NNR-Insuffizienz) nach exogener Gabe von Steroiden diagnostiziert, die sich jedoch mutmaßlich zum Zeitpunkt der Aufnahme bereits wieder erholt hatte, da nach Aufnahme keine Beeinträchtigung der hypothalamisch-hypophysär-kortikotropen Achse mehr nachweisbar war.

## Therapie und Verlauf

Es erfolgte eine antibiotische Therapie mit Cefotaxim, und die Methotrexat-Gabe wurde pausiert. Da der Zustand der Patientin sich rasch besserte, sie am 3. Tag entfiebert war und die Laborwerte für Kortisol und ACTH eine Erholung der Hypophysen-Nebennierenrinden-Achse zeigten, konnte auf eine zunächst diskutierte Stressdosisgabe bzw. Substitutionstherapie mit Hydrocortison verzichtet werden. Bei einer ambulanten Nachkontrolle nach 10 Tagen lag das Kortisol basal mit 2,8 ng/ml noch unter der Norm, bei einem normalen ACTH von 11,7 pg/ml. Auf einen ACTH-Stimulationstest wurde verzichtet. Bei weiterer klinischer Besserung wurde ein abwartendes Vorgehen vereinbart, und ambulante Kontrollen wurden empfohlen.

## Diskussion

Die intraartikuläre Glukokortikoidinjektion ist ein gut etabliertes und bewährtes Verfahren in der pädiatrischen Rheumatologie [18]. Trotz vieler Studien an Erwachsenen, die ein signifikantes postinterventionelles Risiko einer NNR-Insuffizienz belegen [5–9, 11, 13–16, 19, 20], gibt es in der pädiatrischen Literatur nur wenige Daten und ein geringes Bewusstsein für dieses Risiko.

Die bei Weitem häufigste Ursache einer NNR-Insuffizienz stellt die Pharmakotherapie mit synthetischen Glukokortikoiden dar [9]. Unter dieser Therapie kann es zu einer Suppression der Hypothalamus-Hypophysen-Nebennieren-Achse mit Atrophie der kortikotropen Zellen der Hypophyse und der NNR kommen (tertiäre NNR-Insuffizienz). Bei Erwachsenen muss unter einer höher dosierten Pharmakotherapie (Prednisolonäquivalent 20–30 mg täglich) bereits nach wenigen Tagen mit einer Suppression des Regelkreises gerechnet werden [17]. Das Risiko einer NNR-Insuffizienz steigt mit der Dosis und Dauer der Therapie. Auch nach Lokaltherapien muss prinzipiell mit der Entwicklung einer NNR-Insuffizienz gerechnet werden. Daten aus Fallserien belegen das Risiko eines Cushing-Syndroms, aber auch der konsekutiven Störung der Hypothalamus-Hypophysen-Nebennieren-Achse nach einzelnen lokalen Steroidinjektionen [1, 2, 8, 15, 20]. Bei Erwachsenen werden typischerweise 8 h nach intraartikulären Injektionen Spitzenwerte im Serum erreicht. Die lokal verabreichten Glukokortikoide werden innerhalb von 2 bis 3 Wochen vollständig von der Injektionsstelle resorbiert [19]. Das Absorptionsprofil intraartikulärer Glukokortikoide kann je nach Präparat und Injektionsstelle variieren. In entzündeten Gelenken und in stärker vaskularisierten Bereichen wie den Wachstumsfugen ist die Absorption verstärkt.

Wenn das supprimierende Glukokortikoid reduziert oder abgesetzt wird, können Symptome einer NNR-Insuffizienz oder eines Steroidentzuges auftreten. Zu den Symptomen einer NNR-Insuffizienz können Müdigkeit, Übelkeit, Erbrechen, Hypotonie sowie eine erhöhte Infektanfälligkeit gehören [14, 16]. Eine Erholung wird bei den meisten Patienten nach 1 bis 4 Wochen erwartet, kann aber je nach Dosis und Häufigkeit der Injektionen länger dauern [3, 5, 7, 11, 13, 19]. Bei unserer Patientin gehen wir zum Zeitpunkt der stationären Aufnahme im Rahmen des Harnweginfektes von einer Erholung aus, da sich die gemessenen Werte für die hypothalamisch-hypophysär-kortikotrope Achse hier normalisiert hatten, wir gehen jedoch zudem von einer Stressreaktion aus, sodass höhere Werte zu erwarten waren. In einer Studie mit 60 JIA-Patienten wurde ebenfalls eine hohe Prävalenz einer transienten Suppression der Nebenniere gezeigt. Die Speichelkortisolspiegel waren bei 22 Kindern, die eine intraartikuläre Injektion von Triamcinolon-Hexacetonid mit 20–60 mg (meist im Kniegelenk verabreicht) erhielten, über einen Median von 16 Tagen unterdrückt [10]. Keiner der Patienten in dieser Studie entwickelte ein klinisches Cushing-Syndrom, und eine Normalisierung der Kortisolspiegel im Speichel wurde bei der Mehrheit der Patienten vor Ablauf von 4 Wochen beobachtet.

Die NNR-Insuffizienz kann, wenn sie nicht erkannt wird, zu erhöhter Morbidität und Mortalität führen. Eine angemessene „Stressreaktion“, d. h. die Produktion von ausreichend endogenem Kortisol bei akuter Krankheit oder Trauma, kann erheblich beeinträchtigt sein. Bei unserer Patientin könnte sowohl die Verschlechterung des Allgemeinzustandes als auch die Krankheitslast des Harnwegsinfektes durch diese Problematik erklärt werden. In der behandelnden kinderrheumatologischen Einrichtung war trotz großem Kollektiv intraartikulär behandelter Patienten ein derartiger Fall noch nicht aufgefallen. Während die Entwicklung eines Cushing-Syndroms nach lokaler Glukokortikoidtherapie gut belegt ist, finden sich in der Literatur kaum Berichte über gravierende Probleme aufgrund einer temporären Unterdrückung der Hypothalamus-Hypophysen-Nebennieren-Achse bei Kindern nach lokaler Glukokortikoidgabe [12, 20]. Das Risiko eines Steroidentzuges ist allerdings allgemein bekannt. Aus diesem Grund wird die orale Glukokortikoidtherapie üblicherweise langsam ausgeschlichen. Bei intraartikulärer Gabe von Steroidpräparaten ist ein kontrolliertes Ausschleichen nicht möglich, was potenziell zu erheblichen Spitzen mit hohen Kortisolspiegeln in den ersten Wochen nach Injektion und anschließend niedrigen Werten in der Folgezeit führen kann. Diese Problematik ist bei multipler simultaner Anwendung an zahlreichen Körperstellen von noch größerer Relevanz. In einem kürzlich veröffentlichten Umfrageergebnis unter weltweit 358 Kinderrheumatologen wurde die Nebennierenrindeninsuffizienz als mögliche Komplikation nicht dokumentiert. In dieser Umfrage zeigten sich eine große Varianz und geringe Standardisierung der Prozeduren, sowohl was das technische Verfahren, den Einsatz von Ultraschall, die Anästhesie, die Wahl der Medikamente als auch ihre Dosierungen angeht [4]. Es gibt keine evidenzbasierten Empfehlungen zu einer Maximaldosis oder -anzahl bei Mehrfachpunktionen, aber überwiegend werden einzelne oder wenige Gelenke injiziert, der Median lag in der Umfrage bei 4 Gelenken pro Sitzung, aber es gab auch Ausreißer mit einem Maximum von 45 simultan injizierten Gelenken. Der Vorteil der lokalen Glukokortikoidapplikation in Kombination mit einer entlastenden Gelenkpunktion liegt in höheren lokalen Wirkstoffspiegeln an den betroffenen Körperstellen und einem raschen Wirkungseintritt. Ein weiterer Vorteil ist eine längere lokale Wirksamkeit aufgrund der verzögerten Glukokortikoidresorption. Alternativ wäre eine perorale oder intravenöse systemische Brückentherapie mit Glukokortikoiden möglich gewesen. Bei einer peroralen Glukokortikoidtherapie wäre eine Narkoseintervention verzichtbar gewesen. Der Nachteil gegenüber der intraartikulären Therapie ist eine geringere Effektivität sowie eine höhere Tablettenlast mit gastrointestinalen Nebenwirkungen. Auch bei systemischer Gabe von Glukokortikoiden kann es zu einem iatrogenen Cushing-Syndrom kommen, der Hauptvorteil liegt aber in der guten Steuerbarkeit gerade in der Ausschleichphase.

## Fazit

Angesichts der weit verbreiteten Anwendung intraartikulärer Glukokortikoidinjektionen und ihrer klinischen Wirksamkeit müssen sich Ärzte, die diese Injektionen verabreichen, über die potenziellen Risiken des iatrogenen Cushing-Syndroms sowie einer konsekutiven Störung der Hypothalamus-Hypophysen-Nebennieren-Achse im Klaren sein. Patienten und Familien können bei hohen kumulativen Dosen über die Risiken und die Symptome einer Nebennierenrindeninsuffizienz aufgeklärt werden. Die Betreuung der Patienten nach Intervention sollte risikoadaptierten Standards folgen. Patienten können im Bedarfsfall mit oralen Glukokortikoiden supplementiert werden, besonders wenn sie ein Trauma erleiden, sich einem chirurgischen Eingriff unterziehen müssen oder akut erkranken. Weitere Studien im pädiatrischen Patientenkollektiv sollten zu dieser Thematik erfolgen.
